# XbaI and PvuII Polymorphisms of Estrogen Receptor 1 Gene in Females with Idiopathic Scoliosis: No Association with Occurrence or Clinical Form

**DOI:** 10.1371/journal.pone.0076806

**Published:** 2013-10-14

**Authors:** Piotr Janusz, Tomasz Kotwicki, Miroslaw Andrusiewicz, Malgorzata Kotwicka

**Affiliations:** 1 Spine Disorders Unit, Department of Pediatric Orthopedics and Traumatology, University of Medical Sciences, Poznan, Poland; 2 Department of Cell Biology, University of Medical Sciences, Poznan, Poland; University of Iowa Carver College of Medicine, United States of America

## Abstract

**Introduction:**

XbaI single nucleotide polymorphism (SNP) (A/G rs934099) in estrogen receptor 1 gene (*ESR1*) was described to be associated with curve severity in Japanese idiopathic scoliosis (IS) patients and in Chinese patients with both curve severity and predisposition to IS. PvuII SNP (C/T rs2234693) of *ESR1* was described to be associated with the occurrence of IS in the Chinese population; however, two replication studies did not confirm the findings. The *ESR1* SNPs have never been studied in Caucasian IS patients.

**Methods:**

Case-control study. 287 females with IS underwent clinical, radiological and genetic examinations. The patients were divided into three groups according to curve progression velocity: non-progressive IS, slowly progressive IS (progression <1° per month), and rapidly progressive IS (progression ≥1° per month). The radiological maximum Cobb angle was measured and surgery rate established. A control group consisted of 182 healthy females.

**Results:**

All results followed Hardy-Weinberg equilibrium. In the case-control study, genotype frequency in the patients did not differ for the XbaI (AA = 33.5%, AG = 49.1%, GG = 17.4%), nor for the PvuII (TT = 26.8%, TC = 50.2%, CC = 23.0%) comparing to controls (AA = 33.5%, AG = 50.5%, GG = 15.9%) and (TT = 23.1%, TC = 51.1%, CC = 25.8%), respectively, p = 0.3685, p = 0.6046. The haplotype frequency for the patients (AT = 47.1%, GC = 39.2%, AC = 8.9%, GT = 2.8%) did not differ from the controls (AT = 44.8%, GC = 37.4%, AC = 14.0%, GT = 3.8%), p = 0.0645. No difference was found either in XbaI (p = 0.8671) or PvuII (p = 0.3601) allele distribution between the patients and the controls. In the case study, there was no significant difference in genotype frequency for the non-progressive, slowly progressive, and rapidly progressive scoliosis. No difference was found in genotype or haplotype distribution for the mean maximum Cobb angle or the surgery rate.

**Conclusions:**

No association was found between *ESR1* XbaI or *ESR1* PvuII SNP and idiopathic scoliosis in Caucasian females. None of the previously reported associations could be confirmed, regarding curve severity, progression or operation rate.

## Introduction

Idiopathic scoliosis (IS) may occur and progress at any time during childhood and adolescence [1]. It is defined as a three-dimensional spine deformity, which consists of side curve, deviation of sagittal spinal profile and vertebrae rotation in the transverse plane [1]. According to the Scoliosis Research Society, the angle of lateral curvature measured by Cobb`s method on standing antero-posterior radiograph should exceed 10° to make the diagnosis justified [2], [3]. Although IS prevalence is reported to be 2–3% in adolescent population [4], only 10% of the diagnosed children require conservative treatment with corrective bracing and approximately 0.1–0.3% require surgical correction of spinal deformity [1]. The risk of curve progression into severe forms is present until the growth of the spine is completed [4]. Small angle scoliosis progresses more often in girls than in boys [4]. The ability to differentiate between non-progressive and progressive IS is crucial for positive outcomes of early intervention [5].

There exist numerous theories of IS etiology: according to one of the most accepted views, idiopathic scoliosis is a multifactorial disease with an important genetic background [6]. The theory is supported by a higher prevalence of IS in families than in the general population. The occurrence rate was reported at 11.0%, 2.4% and 1.4% for the first, the second and the third degree relatives, respectively [7]. Higher concordance was reported for twins: 73% for monozygotic and 36% for dizygotic twins [8]. Family linkage studies suggested 18 different loci, and over 30 candidate genes were studied for association with IS [9]. Unfortunately, the initial positive results could not be confirmed in the majority of replication studies [9].

Estrogens, being steroid puberty hormones in girls, can act via the Estrogen Receptor type 1 or 2 (ESR1, ESR2) [10] and were suggested to play some role in the pathogenesis of IS [11], [12]. There are two *ESR1* single nucleotide polymorphisms (SNPs), described to reveal a certain association with IS. SNP, rs9340799 A/G, often described as XbaI site polymorphism, has been investigated in the Japanese and the Chinese population.

In 2002, Inoue at al. found association of *ESR1* XbaI site polymorphism with (1) curve severity, (2) the risk of progression of >5° from the initial angle and (3) operation risk in IS Japanese patients [13]. However, in 2011, Takahashi et al. did not confirm the association of this SNP, either with occurrence of IS or curve severity in the Japanese population [14]. XbaI XX (GG) genotype was described in the Chinese Han population by Wu et al. to be associated with occurrence of IS and to be overrepresented in patients with Cobb angle>40° and taller than 160 cm [15]. However, Tang et al. demonstrated no association of XbaI polymorphism, either with occurrence, or with Cobb angle, nor with anthropometric measurements in the Chinese Han population [16]. In 2011, Xu et al. performed a study in the Chinese population and suggested that the X (G) allele at the rs9340799 might be a potential genetic marker, predicting the outcome of brace treatment for IS [17].

The rs2234693 C/T is another SNP of *ESR1*, described as PvuII site polymorphism. Zhao et al. postulated association of PvuII with double curve severe scoliosis in the Chinese population [18]. In three other studies the Chinese or the Japanese population, the PvuII site polymorphism alone did not show any association with IS [13], [15], [16]. Such an association was postulated by Wu et al. in combination with XbaI SNP, therefore, the PpXX (CTGG) might be associated with IS as a “risk” genotype and ppxx (TTAA) as a “protective” genotype [15]. The XbaI and PvuII SNPs are situated 46 bp apart and are in strong linkage disequilibrium [19]. These SNPs can form four different haplotype.

The associations of the XbaI or PvuII site polymorphism with occurrence of IS or curve progression or surgery rate have never been investigated in the Caucasian population.

### Purpose

The aim of this study was to investigate associations of the two *ESR1* SNPs: XbaI and PvuII, with either the occurrence or progression of IS in Caucasian female patients.

## Methods

### Ethics Statement

The study protocol was approved by the Institutional Review Board of the Poznan University of Medical Sciences (No 87/09). All the involved patients – or their parents in case of children – and all volunteers provided written informed consent.

### Patients

From March 2010 till October 2012 two hundred eighty seven Caucasian females with IS, all from one Central European country (Poland), underwent clinical, radiological and genetic examinations. In each case, the age of onset of IS, the age of menarche and treatment history were established. Radiological examination revealed curve pattern, Cobb angle and Risser sign. The Cobb angle under consideration was either the final Cobb angle, defined as the curve angle in skeletally mature patients (N = 253) or Cobb angle, measured in the last follow up of those patients who were skeletally immature (N = 34). Maturity indicators included: (1) the age of at least 16 years, (2) more than 2 years after menarche, (3) Risser sign of 4 or 5 and (4) the end of growth process, defined as height increase of less than 1 cm during previous year. Table 1 contains summary of this data.

**Table 1:** Phenotype description for case-control study.

	Cases	Controls
	N = 287	N = 182
females/males	females	females
age range (years)	10–59	16–64
age mean ±SD (years)	17.9±8.6	28.3±10
menarche	yes or no	yes (more than 2 years after)
family history of scoliosis	positive or negative	negative
back deformity	idiopathic scoliosis (rib hump)	no deformity
curve pattern	all	NA
Cobb angle at final visit	10°–114°	NA
Cobb angle at final visit mean ±SD	39.4°±19.0°	NA
angle of trunk rotation in degrees (Bunell scoliometer)	NA	<4°

NA – non applicable.

### Controls

The control group consisted of 182 healthy Caucasian females. The inclusion criteria for the control group were: (1) the age at least 16 years, (2) more than 2 years after menarche, (3) the angle of trunk rotation of less than 4° on examination with scoliometer, (4) the end of growth process, defined as height increase of less than 1 cm during previous year and (5) negative family history of idiopathic scoliosis. Radiological examination was not performed in the control group. Table 1 contains summary of this data.

### Patients subgrouping

Two separate analyses were performed. First, in a case-control study, an association of SNPs with the occurrence of IS was established for all 287 patients and 182 controls. Second, in a case study of skeletally mature patients, an association of SNPs was established with: (1) the scoliosis progression rate, (2) the mean maximum Cobb angle, (3) moderate (<40°) versus important (≥40°) scoliosis curvature, (4) scoliosis with documented progression of ≥5° Cobb angle versus scoliosis with documented lack of progression, (5) the surgery rate, (6) thoracic versus lumbar scoliosis level and (7) single versus double scoliosis pattern.

For the purpose of analysis of the scoliosis progression rate, the patients were divided into three subgroups, according to the curve progression rate: Non-Progressive IS (NP-IS) with final Cobb angle below 30° (N = 93), Slowly Progressive IS (SP-IS) with the final Cobb angle above 30° but with progression rate below 1°per month (N = 104), Rapidly Progressive IS (RP-IS) with documented radiological progression of ≥1° per month for, at least, 6 months (N = 56). In an analysis of Cobb angle, its maximal values were taken into account. For the purpose of curve size analysis the patients were divided into two subgroups: (1) small or moderate curves with Cobb angle below 40° (N = 161) and (2) important curves with Cobb angle above 40° (N = 92) [15]. For the purpose of surgery rate evaluation surgically treated patients (N = 67) were compared to patients after non surgical therapy (N = 186). Regarding the analysis of scoliosis level, the apical vertebra at the thoracic level (Th2-T11) was considered a criterion for thoracic scoliosis while the apical vertebra at the lumbar level (L2-L4) was a criterion for lumbar scoliosis. Table 2 contains summary of this data.

**Table 2:** Phenotype description for case only study in skeletally matured patients with non-progressive (NP-IS), slowly progressive (SP-IS) and rapidly progressive (RP-IS) idiopathic scoliosis.

	cases N = 253		
phenotype subgroups	NP-IS	SP-IS	RP-IS
	N = 93	N = 104	N = 56
age range (years)	16–52	16–59	12–54
Risser	4 or 5	4 or 5	all
skeletal maturity	yes	yes	no
back deformity	idiopathic scoliosis (rib hump)	idiopathic scoliosis (rib hump)	idiopathic scoliosis (rib hump)
curve pattern	all	all	all
Cobb angle at final visit (range)	10°–30°	30°–65°	35°–114°
Cobb angle at final visit (mean ±SD)	23.3°±5.6°	40.3°±9.2°	69.5°±19.2°
progression rate in degrees per month (range)	NA	0.04°–0.95°	1.0°–3.2°

NA – non applicable.

### Genetic examination

#### Sample collecting

2.7 ml of peripheral blood sample from all the patients and from the controls were collected in S-monovette tubes (SARSTEDT AG & Co., Numbrecht, Germany). A total of 469 samples were studied herein. First, a core group of 287 cases of patients with scoliosis was screened for XbaI and PvuII restriction polymorphism. Then, the second group included 182 non-scoliotic phenotype cases, as a control.

#### DNA isolation

The genomic DNA from each patient was obtained from the collected peripheral blood with Axygen AxyPrep Blood Genomic DNA Miniprep Kit (Axygen Scientific, Inc., Union City, CA, USA) according to manufacturer protocol. DNA concentration was determined spectrophotometrically. 50 to 200 ng of gDNA was used for each PCR reaction.

#### PCR amplification

The 1300 bp and 759 bp fragments of the *ESR1* gene were amplified, using primers described in Table 3. The primers were designed using Primer3Plus online software (http://www.bioinformatics.nl/cgi-bin/primer3plus/primer3plus.cgi/primer3plusAbout.cgi). PCR reactions were carried out in total volume of 25 µl.

**Table 3:** Primers' description.

	Sequence 5′–>3′	annealing temp	amplicon length
ESR1_rs9340799_XbaI_F	CTGCCACCCTATCTGTATCTTTTCCTATTCTCC	71°C	1300 bp
ESR1_rs9340799_XbaI_R	TCTTTCTCTGCCACCCTGGCGTCGATTATCTGA		
ESR1_rs2234693_PuvII_F	AGGCTGGGCTCAAACTACAG	60°C	759 bp
ESR1_rs2234693_PuvII_R	TCCTTGGCAGATTCCATAGC		

The reaction mixture for the first *ESR1* gene fragment contained in final concentration: 50 ng of template DNA, 1x KAPA HiFiHotStartReadyMix Polymerase (containing 2.5 mM Mg^2+^; KapaBiosystems, Boston, MA, USA) and 0.3 µM of each primer (Genomed, Gdansk, Poland).

The reaction mixture for the second *ESR1* gene fragment included (final concentration): 200 ng of gDNA, 1x OptiBuffer, 3.5 mM MgCl_2_ solution, 300 nM of each primer, 400 nM of each dNTP, 0,5% DMSO and 1U of BIO-X-ACT DNA polymerase. Compounds used to amplify the second *ESR1* DNA fragment were obtained from Bioline GmbH, Germany.

The thermal profiles for each fragment were polymerase's and primer's depended and they are shown in Table 4.

**Table 4:** PCRs reactions thermal profiles.

Step	ESR1 – first amplicon		ESR1 – second amplicon	
	duration	cycles no.	duration	cycles no.
Initial denaturation	95°C, 5 min	1	95°C, 5 min	1
Denaturation	95°C, 20 sec	30	95°C, 30 sec	40
Annealing	71°C, 15 sec	30	60°C, 30 sec	40
Elongation	72°C, 15 sec	30	70°C, 1.20 sec	40
Final elongation	72°C, 3 min	1	70°C, 5 min	1

The 2 µl of the PCR reaction products were electrophoresed on 2% agarose gel (FMC BioProducts, Rockland, ME, USA) in the presence of ethidium bromide to confirm its identity with molecular mass marker. For 10% of the randomly selected samples half of the reaction mixture were purified according to AxyPrepPCR Clean-up Kit manufacturer's protocol and sequenced to confirm its identity to the sequences deposited in NCBI gene sequences database. All experiments were replicated twice.

#### Restriction Fragments Length Polymorphism (RFLP) analysis

The 1.5 µl of each PCR reaction was used for *XbaI* and *PvuII* restriction analysis. SNPs polymorphisms for each fragment were described in Table 5.

**Table 5:** RFLP SNPs fragment description.

	recognition site	Allele	digestion product length
ESR1_rs9340799_XbaI	T*CTAGA	T*CTAGA	910bp +390bp
		TCTGGA	1300bp
ESR1_rs2234693_PuvII	CAG*CTG	CAG*CTG	271bp +488 bp
		CAGCCG	759 bp

* digestion site.

The digestion was conducted in total volume of 15 µl mixture contained: 1.5 µl of the PCR product, 1 µl FastDigest Green Buffer, 1FDU of the FastDigestEnyzme (*XbaI* or *PuvII*) and DNase free water. The Restriction reaction was incubated in thermocycler 1 hr at 37°C and followed by thermal inactivation of the restriction enzyme at 85°C, 10 min. Afterwards the 10 µl of the reaction were electrophoresed on 2% agarose gel in the presence of ethidium bromide to establish the restriction's allele. The results were described as AA or GG or AG for *XbaI* and CC or TT or CT for heterozygotic allele for *PuvII*.

### Statistical evaluation

The Chi^2^ test was used to determine the differences in allele, genotype and haplotype distribution for the patients with IS, the controls and all the patients subgroups. All archived genotypes were tested for the Hardy-Weinberg equilibrium with Chi^2^ test. A comparison of the mean Cobb angle among the three patients subgroups was verified with the Kruskal-Wallis test. The calculation was made with GraphPad StatMate 2.0. An analysis of the linkage disequilibrium of two polymorphisms was performed with support of the CobeX web tool [20]. Haplotype evaluation was done with the WinHAP program ver. 1.0 [21]. The calculation of the required sample size, based on genotypes frequencies in Central European population revealed that a sample size of 282 cases and 178 controls had 80% power to detect odds ratio (OR) of 1.68 (previously reported OR for the XbaI polymorphism [15]) with a significance level of 0.05 two-tailed. The power calculation for the association study was performed with the G*Power 3.1.7. For all statistics the P value of 0.05 and CI 95% were considered significant.

## Results

The genotype distribution for the patients, the controls and all the subsequent groups did not deviate from the Hardy-Weinberg equilibrium.

The XbaI and PvuII site polymorphisms showed strong linkage disequilibrium, D` = 0.838 and r^2^ = 0.5092 with p<0.0001.

### Case-control study: an association of allele and genotype with predisposition to IS

No significant differences were found, either in allele or genotype frequency between IS patients and healthy controls (Table 6).

**Table 6:** Allele and genotype frequency for IS patients and healthy controls.

SNP			IS patients N = 287	HWE	Healthy controls N = 182	HWE	p value a dominant^b^	p value a recessive^c^	p value a allele de-pendent	Odds ratio (95% CI)
XbaI	Genotype	AA	33.5% (96)		33.5% (61)					
		AG	49.1% (141)	0.89	50.5% (92)	0.56	0.7696	0.9880	0.3685	0.90 (0.54–1.48)**
		GG	17.4% (50)		15.9% (29)					0.99 (0.67–1.48)***
	Allele	A	58.0% (333)		58.8% (214)					
		G	42.0% (241)		41.2% (150)				0.8671	0.97 (0.74–1.26)
PvuII	Genotype	TT	26.8% (77)		23.1% (42)					
		TC	50.2% (144)	0.93	51.1% (93)	0.76	0.5572	0.4230	0.6046	1.17 (0.76–179)**
		CC	23.0% (66)		25.8% (47)					1.22 (0.79–1.88)***
	Allele	T	51.9% (298)		48.6% (177)					
		C	48.1% (276)		51.4% (187)				0.3601	0.14 (0.87–1.48)

a Chi^2^ test.

b Dominant model contrasts: for XbaI XX and Xx vs xx, for PvuII PP and Pp vs pp.

c Recessive model contrasts: for XabI XX vs Xx and xx, for PvuII PP vs Pp and pp.

There was no significant difference in haplotype frequency between IS patients and healthy controls (Table 7).

**Table 7:** Haplotype frequency for IS patients and healthy controls.

	Haplotype				
	AT^a^	AC^a^	GT^a^	GC^a^	p value^b^
Patients	49.1% (282)	8.9% (51)	2.8% (16)	39.2% (225)	
Controls	44.8% (163)	14.0% (51)	3.8% (14)	37.4% (136)	0.0645

a calculated with WinHAP [21].

b Chi^2^ test.

### Case study: an association of allele and genotype with selected parameters of IS

#### Association of genotype with progression rate

The distribution of allele and genotype in rapidly progressive versus slowly progressive versus non-progressive idiopathic scoliosis did not show statistically significant differences (Table 8).

**Table 8:** Allele and genotype frequency for the patients with non-progressive (NP-IS), slowly progressive (SP-IS) and rapidly progressive (RP-IS) idiopathic scoliosis (N = 253).

SNP		NP-IS		SP-IS		RP-IS		p value a dominant^b^	p value a recessive^c^	p value a allele de-pendent
		N = 93		N = 104		N = 56				
		% (n)	HWE	% (n)	HWE	% (n)	HWE			
XbaI	AA	38.7% (36)		28.9% (30)		33.9% (19)				
	AG	41.9% (39)	0.21	56.7% (59)	0.11	42.9% (24)	0.32			
	GG	19.4% (18)		14.4% (15)		23.2% (13)		0.3629	0.5498	0.2194
PvuII	TT	24.7% (23)		26.9% (28)		30.4% (17)				
	TC	53.8% (50)	0.46	52.9% (55)	0.52	37.5% (21)	0.06			
	CC	21.5% (20)		20.2% (21)		32.1% (18)		0.2041	0.7546	0.2830

a Chi^2^ test.

b Dominant model contrasts: for XbaI XX and Xx vs xx, for PvuII PP and Pp vs pp.

c Recessive model contrasts: for XabI XX vs Xx and xx, for PvuII PP vs Pp and pp.

There was no significant difference in haplotype frequency among the patients with rapidly progressive, slowly progressive and non-progressive idiopathic scoliosis (Table 9).

**Table 9:** Haplotype frequency for the patients with non-progressive (NP-IS), slowly progressive (SP-IS) and rapidly progressive (RP-IS) idiopathic scoliosis.

	Haplotype				
	AT^a^	AC^a^	GT^a^	GC^a^	p value b
NP-IS	48.9% (91)	10.8% (20)	2.7% (5)	37.6% (70)	
SP-IS	50.5% (105)	6.7% (14)	2.9% (6)	39.9% (83)	0.8482
RP-IS	45.5% (51)	9.8% (11)	3.6% (4)	41.1% (46)	

a calculated with WinHAP [21].

b Chi^2^ test.

#### Association of genotype with IS severity

The mean maximum Cobb angle in skeletally mature patients did not significantly differ among genotype (XbaI p = 0.8475, PvuII p = 0.1499) (Table 10). For the patients, divided by Cobb angle into two subgroups: moderate below 40° and important above 40°, no significant difference in genotype distribution was found (XbaI p = 0.7434, PvuII p = 0.1170) (Table 10). There was no significant difference of genotype distribution in scoliosis with documented progression of Cobb angle ≥5° versus scoliosis with documented lack of progression (XbaI p = 0.5178, PvuII p = 0.1800). There was no difference of genotype distribution between surgically treated and non surgically treated patients (XbaI p = 0.8052, PvuII p = 0.1637) (Table 10).

**Table 10:** Genotype according to Cobb angle, progression or surgical treatment (N = 253).

	XbaI (rs9340799)			p value	PvuII (rs2234693)			p value
Genotype	AA	AG	GG		TT	TC	CC	
Number of subjects	85	122	46		68	126	59	
Mean Cobb angle (±SD)	38.7±21.2	38.6±18.8	38.1±18.0	0.8475^a^	40.9±19.7	36.4±19.2	40.4±19.5	0.1499^a^
Cobb ange<40°	34.1% (55)	49.1% (79)	16.8% (27)		24.8% (40)	41.3% (88)	20.5% (33)	
Cobb ange≥40°	32.6% (30)	46.7% (43)	20.7% (19)	0.7434^b^	30.4% (28)	41.3% (38)	28.3% (26)	0.1170^b^
Documented progression >5°	31.1% (41)	48.5% (64)	20.4% (27)		27.3% (36)	44.7% (59)	28.0% (37)	
Documented lack of progression	36.4% (44)	47.9% (58)	15.7% (19)	0.5178^b^	19.0% (23)	55.4% (67)	25.6% (31)	0.1800^b^
Patients undergoing surgery	35.8% (24)	44.8% (30)	19.4% (13)		29.9% (20)	40.2% (27)	29.9% (20)	
Patients without surgery	32.8% (61)	49.5% (92)	17.7% (33)	0.8052^b^	25.8% (48)	53.2% (99)	21.0% (39)	0.1637^b^

a Kruskal-Wallis test,.

b Chi^2^ test.

#### Association of genotype with IS curve level and curve pattern

Neither the vertebral level of the main curve apex – thoracic versus lumbar (XbaI p = 0.3525, PvuII p = 0.8466), nor the numbers of structural curves – single versus double (XbaI p = 0.5261, PvuII p = 0.8502) did show any association with genotype (Table 11).

**Table 11:** Genotype according to curve level and curve pattern (N = 253).

	XbaI (rs9340799)			p value	PvuII (rs2234693)			p value^a^
Genotype	AA	AG	GG		TT	TC	CC	
Main thoracic curve	33.0% (59)	50.8% (91)	16.2% (29)		27.4% (49)	50.3% (90)	22.3% (40)	
Main lumbar curve	35.1% (26)	41.9% (31)	23.0% (17)	0.3525	25.7% (19)	48.6% (36)	25.7% (19)	0.8466
Single curve	37.5% (39)	46.2% (48)	16.3% (17)		26.0% (27)	51.9% (54)	22.1% (23)	
Double curve	30.9% (46)	49.7% (74)	19.4% (29)	0.5261	27.5% (41)	48.3% (72)	24.2% (36)	0.8502

a Chi^2^ test.

#### Association of haplotype combination with IS severity

There were no associations of patients haplotype distribution with curve severity, progression or surgery rate (Table 12).

**Table 12:** Haplotype according to Cobb angle, progression or surgical treatment.

	Haplotype				
	AT^a^	AC^a^	GT^a^	GC^a^	p value^b^
Patients with Cobb <40°	49.4% (159)	9.3% (30)	2.8% (9)	38.5% (124)	
Patients with Cobb ≥40°	47.8% (88)	8.2% (15)	3.2% (6)	40.8% (75)	0.9248
Documented progression >5°	46.2% (122)	9.1% (24)	4.2% (11)	40.5% (107)	
Documented lack of progression	51.6% (125)	8.7% (21)	1.7% (4)	38.0% (92)	0.2976
Patients undergoing surgery	47.8% (64)	10.4% (14)	2.2% (3)	39.6% (53)	
Patients without surgery	49.2% (183)	8.3% (31)	3.2% (12)	39.3% (146)	0.8344

a calculated with WinHAP [21].

b Chi^2^ test.

#### Association of haplotype combination with IS curve level and curve pattern

There were no association of patients haplotype distribution with number of the curves or the vertebral level of the main curve (Table 13).

**Table 13:** Tabel 13. Haplotype according to curve level and curve pattern.

	Haplotype				
	AT^a^	AC^a^	GT^a^	GC^a^	p value^b^
Main thoracic curve	50.6% (181)	7.8% (28)	2.0% (7)	39.6% (142)	
Main lumbar curve	44.6% (66)	11.5% (17)	5.4% (8)	38.5% (57)	0.0864
Single curve	50.9% (106)	9.6% (20)	1.0% (2)	38.5% (80)	
Double curve	47.3% (141)	8.4% (25)	4.4% (13)	39.9% (119)	0.1455

a calculated with WinHAP [21].

b Chi^2^ test.

## Discussion

The genotype frequency of XbaI and PvuII in 182 controls was comparable with the data, stored in Hap Map CEU (Utah residents with Northern and Western European ancestry) and with publications concerning Caucasian populations: no statistically significant difference in genotype distribution was found (Table 14).

**Table 14:** Comparison of genotype frequency in 182 controls in this study versus controls from other studies concerning Caucasian populations.

	N	XbaI (rs9340799)			p value a	PvuII (rs2234693)			p value a
		AA	A/G	GG		TT	C/T	CC	
Hap Map CEU	59	29	25	5	0.0711	28	58	17	0.1889
Becherini et al. [19] Italy	610	213	280	117	0.4664	177	299	134	0.2437
Patel et al. [22] Canada	662	273	307	82	0.1324	197	333	132	0.1003
Molvarec et al. [23] Hungary	180	60	93	27	0.9637	45	94	41	0.7761
Ignaszak-Szczepaniak et al. [24] Poland	163	65	84	14	0.0958	42	78	43	0.7991
Stavrou et al. [25] Greece	145	54	56	35	0.0599	41	53	51	0.0296
Roszkowska-Gancarz et al [26] Poland	414	157	194	63	0.5852	118	199	97	0.3823

a Chi^2^ test.

In this study, two analyses were consecutively performed. First, an association of XbaI and PvuII site polymorphisms with the occurrence of IS was established for 287 patients and 182 controls. Second, an association with selected clinical or radiological parameters was studied for 253 patients, who achieved skeletal maturity: in those patients, the final Cobb angle could be established at the end of growth process. In contrast to the observation published by Wu et al. [15], we did not find any significant difference.

in XbaI genotype distribution between females with IS and healthy controls. No overrepresentation of the G allele (X) or the CTGG haplotype (PpXX) was found in the study group. No association of the ESR1 gene polymorphism could be established with either Cobb angle, progression, surgery, curve pattern or curve number. Our results are consistent with the data, published by Tang et al. [16] for the Chinese population and with the publication by Takahashi et al. [14] for the Japanese population. Moreover, we could not confirm the findings of the case only study by Inoue et al. [13] concerning the association of the SNP with curve severity, the risk of progression or the surgery rate.

Taking into consideration this study sample size as well as previously reported data concerning the SNPs risk, it was found that for the OR = 2.0 the power achieved is 95% and for the reported OR = 1.68 the power is 80% [15].

In this study, much attention was paid to precise phenotype evaluation in the patients. The division of natural course scoliosis into NP-IS, SP-IS and RP-IS subgroups was based on clinical relevance, as proposed by Dimeglio [27]. Early detection of RP-IS patients is crucial for effective treatment outcome [28]. On the other hand, according to Weinstein et al. [29], scoliosis with Cobb angle below 30° does not affect patient`s quality of life and remains stable throughout the lifetime without tendency for progression [29]. This clinical form was named in our study Non-Progressive IS (NP-IS).

Taking into consideration gender shift, demonstrated by a higher progression rate in adolescent females, in association with puberty, sex hormones may have influence on curve progression [4]. That is why genetic studies in the *ESR1* area are justified. The PvuII and XbaI site polymorphisms are located in intron 1, 46 bp apart, of the ESR1 gene. It is postulated that the SNPs do not exert any direct impact on the receptor structure [30], however a regulatory role of the SNPs was suggested, as a potential enhancer of gene expression [31]. The XbaI and Pvu II SNPs were described to be associated with the age of menarche. Stavrou et al. described a later onset of menarche in patients with XbaI XX (GG) genotype and with XP (GC) haplotype [25]. Delayed hormonal maturity may be associated with longer period of risk of progression in IS and bigger final Cobb angle [32]. SNPs association with bone mineral density (BMD) was described [30], the data being, however, incoherent [33]. Low BMD values in IS were indicated in many studies [34] as a possible linkage between *ESR1* polymorphism and IS. Estrogen receptor is expressed both in osteoblasts and osteoclasts [35], [36].

## Conclusions

In the Central European population, no association was found between *ESR1* XbaI and PvuII site polymorphisms and IS, nor any of the previously reported associations was confirmed, regarding the surgery rate, curve severity or curve progression.
